# Caregiver Burden among Caregivers of Patient Undergoing Hemodialysis in Tertiary Care Center : A Descriptive Cross-sectional Study

**DOI:** 10.31729/jnma.4779

**Published:** 2020-03-31

**Authors:** Srijana Khatri Chhetri, Rojina Baral

**Affiliations:** 1 Department of Nursing, College of Medical Sciences Bharatpur, Chitwan, Nepal

**Keywords:** *burden*, *caregiver*, *hemodialysis*

## Abstract

**Introduction:**

Chronic kidney disease affects almost all aspects of life of the patients and caregivers. Dialysis is a common treatment modality for chronic renal failure. The caregivers of patients undergoing hemodialysis bear the emotional and psychological stress of having a chronically ill patient. The physical and psychological distress, limitations in personal and social activities, loss of freedom, financial limitations directly or indirectly affect the level of burden among caregivers. This study aims to study the level of burden among caregivers of patient undergoing hemodialysis.

**Methods:**

A descriptive cross-sectional study among 123 caregivers giving care to hemodialysis patients for at least 3 months at Teaching Hospitals, Chitwan was carried out using simple random sampling technique. Level of burden was evaluated using the burden questionnaire (Zarit Burden Interview).

**Results:**

The study revealed that 60 (48.78%) had mild to moderate, 53 (43.08%) had moderate to severe. The median scores for burden among the caregivers was (39.30±11.68) with 44.65%.

**Conclusions:**

Coping Strategies, social support, support interventions has greater impact on caregivers in achieving their roles in caring the patients and increases the capability to cope effectively with the patient's condition.

## INTRODUCTION

Hemodialysis is a treatment modality which contributes to the better physical condition of the patient by preventing further complications due to uremia.^1^ According to Global Burden of Disease Study, kidney disease was the 12th most common cause of death, accounting for 1.1 million deaths worldwide^2^ and as per World health ranking the death rate in Nepal in case of kidney disease is 21.72 per 100,000 and Nepal falls in 52nd rank among 183 countries.^3^

Patients on dialysis require caregiving and assistance in their daily lives from family members and others for hospital visitation and supervised administration which places a considerable burden on caregivers.^4^ The family burden is mainly caused by the combination of physical work, emotional pressure, social restrictions, and economic demands during the provision of care to their patients and this has been found to be associated with a significant reduction in caregivers' quality of life and their health status.^5^ Therefore, this study aims to study the level of burden among caregivers of patient undergoing hemodialysis.

## METHODS

A descriptive cross-sectional study was carried among caregivers of patient undergoing hemodialysis at two Teaching Hospitals, Chitwan. Ethical Clearance was obtained from the Institutional Review Committee of College of Medical Sciences Teaching Hospital (COMSTH). Written consent was obtained from each sample before collecting data. Data was collected by using Simple random sampling technique. Random number table was used to collect a total of 123 samples. Sample size was calculated using the formula

n= Z^2,^ × p × q/e^2^

where Z= 1.96 at 95%, confidence interval
         p= prevalence, 49.4%^13^
         q= 1-p
         e= margin of error, 5%
n= 155 and Non-response rate= 10%.

And using Probability Proportion Size, 69 sample from COMS-TH and 54 samples from CMC-TH was collected. The data was collected from 12th May to 10th June, 2019 from both tertiary care center. Caregivers providing care to hemodialysis patients for at least 3 months and with no severe life events within past 3 months were selected for the study. Zarit Burden Interview was used as a tool to collect the data. Permission from Mapi Research Trust was obtained and validated version of Zarit Burden Interview in Nepali language was used for the study. Data obtained was reviewed, coded and entered in SPSS 23 version and the descriptive statistical analysis was done.

## RESULTS

Among 123 caregivers, 71 (57.72%) were female and remaining were male. Maximum participants 48 (39.02%) were of age 20-39 years whereas 6 (4.87%) were of age =19 years. Majority of the participants 52 (42.27%) were illiterate, and among literates 32 (26.01%) were having secondary level education. Most of the participants 69 (56.09%) of them were spouse of patients. Similarly, 58 (47.15%) of caregiver's time period of giving care to patient was more than 3 years. Also 51 (41.46%) of the patients were hypertensive and 58 (47.15%) of patient's functional status was poor (Table 1 and 2).

**Table 1:** Caregiver's Socio-demographic Characteristics.

S.N.	Demographic Variables	n(%)
1.	Caregiver's Age	
	< =19 years	6 (4.87)
	20 - 39 years	48 (39.02)
	40 - 59 years	46 (37.39)
	>=60 years	23 (18.69)
2.	Sex	
	Male	52 (42.27)
	Female	71 (57.72)
3.	Education	
	Illiterate	52 (42.27)
	Primary level education	19 (15.44)
	Secondary level education	32 (26.01)
	Higher secondary level education	13 (10.56)
	Intermediate of post school Diploma	2 (1.62)
	Graduate or postgraduate	2 (1.62)
	Professional	3 (2.43)
4.	Marital status	
	Single	16 (13)
	Married	103 (83.73)
	Widowed	1 (0.81)
	Divorced/Separated	3 (2.43)
5.	Occupation	
	Unemployed	58 (47.15)
	Unskilled worker	3 (2.43)
	Semi-skilled	3 (2.43)
	Skilled worker	56 (45.52)
	Clerk, Shop owner, Farm	1 (0.81)
	Profession	2 (1.62)
6.	Type of family	
	Nuclear	64 (52.03)
	Joint	59 (47.96)
7.	Relationship with patient
	Child	14 (11.38)
	Parent	36 (29.26)
	Sibling	4 (3.25)
	Spouse	69 (56.09)
8.	Monthly income of the family	
	<=Rs.2300	25 (20.32)
	Rs.2301-Rs.6850	9 (7.31)
	Rs.6851-11450	5 (4.06)
	Rs.11451-Rs.17150	17 (13.82)
	Rs.17151-Rs.22850	15 (12.19)
	Rs.22851-Rs.45750	32 (26.01)
	<=Rs.45751	20 (16.26)
9.	Duration of Caregiving
	3 months to 6 months	19 (15.44)
	6 months to 1 year	10 (8.13)
	1 year to 3 years	36 (29.26)
	More than 3 years	58 (47.15)
10.	Monthly Expenditure for Treatment	
	Rs. 10000 -20000	66 (53.65)
	Rs. 21000 - 30000	45 (36.58)
	Rs. 31000 - 40000	8 (6.50)
	>= Rs. 40000	4 (3.25)

**Table 2:** Patient's Socio-demographic Characteristics.

S.N.	Demographic Variables	n(%)
1.	Patient's Age	
	<=19 years	4 (3.25)
	20 - 39 years	27 (21.95)
	40 - 59 years	47 (38.21)
	>=60 years	45 (36.58)
2.	Sex	
	Male	70 (56.91)
	Female	53 (43.08)
3.	Duration of Illness	
	3 months to 6 months	13 (10.56)
	6 months to 1 year	9 (7.31)
	1 year to 3 years	33 (26.82)
	More than 3 years	68 (55.28)
4.	Duration of Hemodialysis	
	3 months	12 (9.75)
	3 months to 6 months	24 (19.51)
	6 months to 1 year	15 (13)
	More than 1 year	72 (58.53)
5.	Comorbidities	
	Hypertension	51 (41.46)
	Diabetes	8 (6.50)
	Cardio Vascular Diseases	8 (6.50)
	Both A & B	29 (23.57)
	Others	17 (13.82)
	No any comorbidities	10 (8.31)
6.	Functional Status	
	Usually do with no difficulty	5 (4.06)
	Does with some difficulty	51 (41.46)
	Does with more difficulty	9 (7.31)
	Usually do not do because of health condition	58 (47.15)

**Figure 1:**
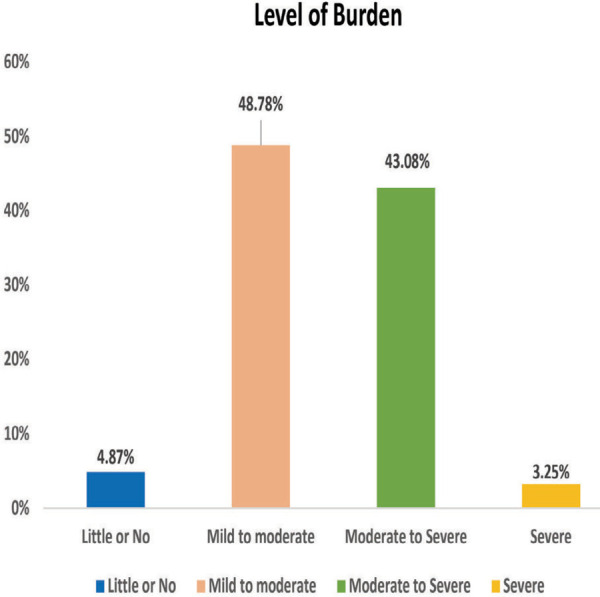
Diagram showing percentage of caregivers' level of burden.

Majority of caregiver 60 (48.78%) had mild to moderate burden and 53 (43.08%) had moderate to severe burden (Figure 1). The overall mean burden score was 39.30±11.68.

## DISCUSSION

Caregivers of patient receiving hemodialysis feel more responsibilities and are at higher risk of developing emotional and psychological distress, low quality of life and increased burden. In spite of their daily obligations, they tend to fulfill the need of patients which sums up with the feeling of burden.

In this study majority of the caregivers are females (57.72%). Since women being more willing to embrace caregiving roles can be attributed to women being more compassionate and emotional and being better in coping with the difficulties of caregiving. This finding is comparable to the finding of the study conducted in Turkey where majority of the caregivers were female.^6^ Similarly, the majority of the caregivers (56.09%) were spouse in the relationship to the patient which is consistent to a study conducted in China which reveals that 93% of the caregivers were spouses.^7^ Maximum 47.15% of the caregivers belongs to more than 3 years of caregiving duration. It can be attributed as, the longer the caregivers took care of their patients, the greater burden they endured and the poorer mental health they had.^8^ In this study majority of the patients are older (≥60 yrs) which implies the patient to involve less in physical activities and are more likely to seek care from caregivers. Similar findings have been suggested by the study conducted in Jordan.^9^

In this study, most of the caregivers perceived mild to moderate burden (48.78%). This findings were supported by the study conducted in Rawalpindi, Pakistan where 65% of the caregivers perceived mild to moderate burden.10 Studies done in different Asian countries also demonstrate similar findings.^11,12^

## CONCLUSIONS

Caregivers may develop emotional and psychological distress, low quality of life, financial problems, increased workload, anger depression, a feeling of helplessness and then to an increased burden. Therefore, Coping Strategies, social support, support interventions should be planned to assist the caregivers in achieving their roles in caring the patients and increases the capability to cope effectively with the patient's condition.
